# Role of handedness-related vestibular cortical dominance upon the vestibular–ocular reflex

**DOI:** 10.1007/s00415-015-7690-y

**Published:** 2015-03-06

**Authors:** Q. Arshad, M. Patel, U. Goga, Y. Nigmatullina, A. M. Bronstein

**Affiliations:** Division of Brain Sciences, Academic Department of Neuro-otology, Imperial College London, Charing Cross Hospital Campus, Fulham Palace Road, London, W6 8RF UK

Dear Sirs,

Cortical influences over low-order vestibular function such as the vestibular–ocular reflex (VOR) are widely accepted [1–3]. Hallpike and colleagues originally demonstrated that patients with temporal lobe lesions, exhibit a strong asymmetry (i.e. “directional preponderance”), in the vestibular nystagmus elicited during caloric stimulation” [3]. Recent work to establish the neural correlates of human vestibular cortical processing have implemented three main approaches. Functional imaging [4–6], clinical lesion studies [2, 7, 8] and brain stimulation data [9–11] have all implicated tempo-parietal areas, usually with right hemisphere dominance. However, it was not until the seminal paper by Dieterich et al. [4] that the concept of handedness-related vestibular hemispheric dominance took shape, showing that the right hemisphere is vestibular dominant in right-handed individuals and vice versa in left handers.

In this context, we read with interest the letter published on the 28th of October in this journal by Brandt and Dieterich, in which they state that vestibular dominance is concerned solely with “higher” but not “lower” order vestibular functions [12]. Here we firstly review our previously published data and secondly present new findings, which in contradiction to Brandt and Dietrich’s statement, demonstrates that handedness-related vestibular dominance is linked to “lower-order” functions such as the VOR.

**Fig. 1:**
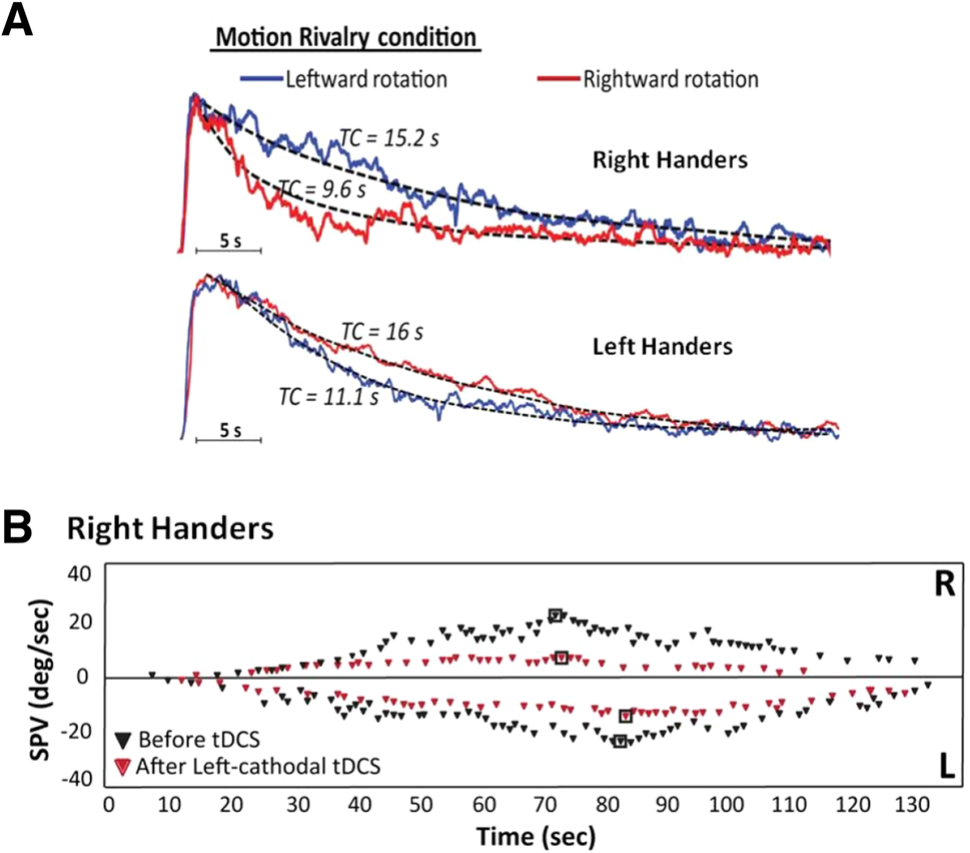
**a** Modified from Arshad et al. [13, 14]. Handedness-related cortical modulation of the vestibular–ocular reflex. J Neurosci 33:3221–3227. *Data curves* represent (*post-rotational*) grand average slow phase eye velocity (SPV) following either rightward rotation (*red curve*) or leftward rotation (*blue curve*) and concurrent binocular rivalry viewing. The *black dotted lines* are exponential fitted curves to measure the time constant of decay (TC) of the post-rotational VOR. As shown, in right handers the VOR is suppressed following rightward rotation (i.e. *red curve*) but following leftward rotation (i.e. *blue curve*) in left handers. **b** Modified from Arshad et al. [11]. Left cathodal trans-cranial direct current stimulation of the parietal cortex leads to an asymmetrical modulation of the vestibular–ocular reflex. Brain Stimul 7:85–91. A representative *trace* showing how caloric responses are suppressed following left cathodal stimulation in a right-handed subject. The *dots* represent slow phase eye velocities over time, before (*black dots*) and after (*red dots*) left cathodal tDCS. The *black square* denotes the peak SPV. *Top panel* represents the responses for right ear cold irrigations, with the *bottom panel* representing the response to left ear cold irrigations. Note the *marked* suppression of vestibular nystagmus following left cathodal stimulation, and that a greater degree of suppression is observed for right compared to left ear cold irrigations

Initially, we demonstrated for the first time behaviourally (previous studies were fMRI based) [4–6] a handedness-related cortical modulation of the VOR [13]. We rotated (i.e. velocity step 90^o^/s) subjects on a motorised Barany chair, whilst either viewing bi-stable visual percepts (i.e. motion binocular rivalry) or performing a visuo-spatial working memory task, and found that the post-rotational VOR was asymmetrically suppressed. Notably, the directionality of the asymmetrical modulation was related to handedness. That is, in right handers we observed that the post-rotational VOR (i.e. stopping response) was suppressed following rightward rotations (left-beating vestibular nystagmus). Conversely, in left handers we observed that the post-rotational VOR was suppressed following leftwards rotation (right-beating nystagmus) [13] (Fig. 1a).

To probe the neural mechanisms underpinning such asymmetrical suppression, we modulated parietal cortex excitability using trans-cranial direct current stimulation (tDCS). We measured the VOR elicited via cold water (30 ^o^C) caloric irrigations which induce a vestibular nystagmus that beats towards the non-stimulated ear, before and after tDCS in right-handed subjects. Facilitation of the right hemisphere (anodal stimulation) and concurrent inhibition of the left hemisphere (cathodal stimulation) resulted in a suppressed VOR during right ear cold irrigations (left-beating nystagmus) but normal responses during left ear cold irrigations (right-beating nystagmus). To identify the active electrode, we subsequently applied unipolar tDCS and found that cathodal stimulation of the left hemisphere resulted in a bilateral but asymmetrical suppression of the VOR. That is, right ear cold irrigations that previous fMRI studies have shown to be predominantly processed by the left hemisphere were suppressed to a greater degree than left ear cold irrigations which are predominantly processed by the right hemisphere (Fig. 1b) [5, 6, 11]. We previously proposed that the differential effects (i.e. unilateral as opposed to bilateral suppression) observed upon the VOR in the two different tDCS montages was most likely attributable to the facilitation of the right hemisphere during bi-hemispheric stimulation [11].

**Fig. 2:**
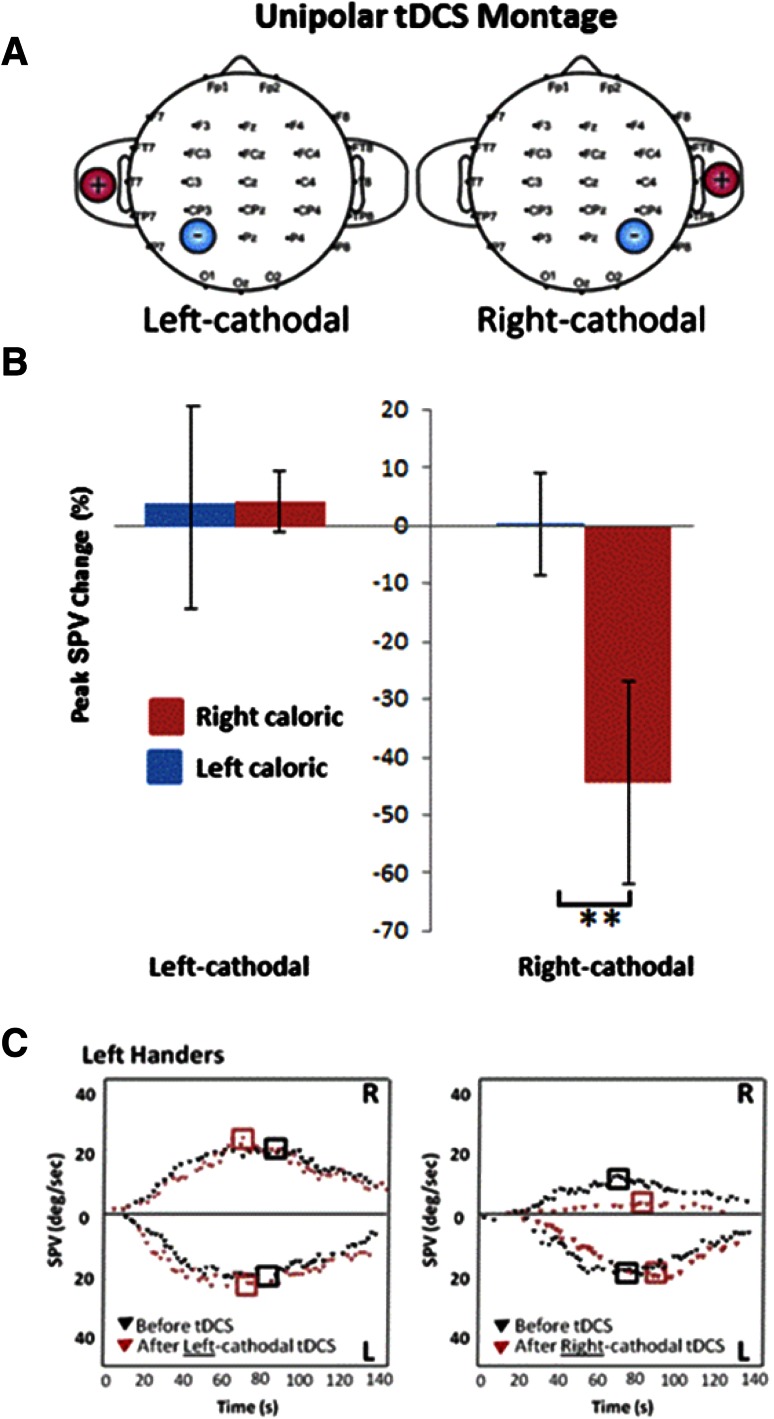
**a** Unipolar tDCS montage implemented for left-handed subjects for the work reported here. This montage was applied for either left (P3: international 10–20 system for EEG electrode placement; electrode placement area 25 cm^2^) or right (P4) hemisphere cathodal stimulation. The reference electrode was placed on the ipsilateral shoulder. Stimulation was applied using a battery driven stimulator (neuroConn, GMBH, Ilmenau, Germany). A constant 1.5 mA current was employed for 15 min, with a ramp up and fade out time of 10 s. **b** Following right cathodal stimulation (*right panel*) a marked suppression in the peak SPV is observed for right but not left ear cold irrigations. Following left cathodal stimulation (*left panel*) no change in peak SPV was observed during either right or left ear cold irrigations. Data marked ** is significant at *p* < 0.001. **c** Shows a representative VOR response (SPV plotted over time) from a single subject following either left cathodal (*left panel*) or right cathodal (*right panel*) stimulation. The SPV are shown in *black* before tDCS and in red following tDCS. *Black squares* represent the peak SPV. Following left cathodal stimulation (*left panel*) no effect is observed upon the VOR during either right or left ear cold irrigations. Following right cathodal stimulation (*right panel*) left ear caloric irrigations remain unaffected, however, note the marked suppression in the VOR for right ear cold caloric irrigations

Accordingly, we predicted that in left-handed subjects, inhibition of the right not the left hemisphere (Fig. 2a) should induce an asymmetrical modulation of the VOR. Herewith, we report that in six left-handed subjects we found this to be the case, specifically for those caloric irrigations that in left handers are predominantly right hemisphere based (right ear cold irrigations (30 ^o^C) [5, 6] (Fig. 2b, c). A 2 × 2 repeated-measures ANOVA revealed a significant main effect for side of caloric [*F* (1,5) = 39.85, *p* < 0.001), time (i.e. pre-post tDCS) [*F* (1,5) = 25.74, *p* < 0.004 and a significant interaction for side*time [*F* (1,5) = 37.29, *p* < 0.002). Post hoc paired *t* tests (Bonferroni corrected) revealed significant differences for right but not left ear cold irrigations following right cathodal stimulation (*p* < 0.001, *t* = 9.8 and *p* > 0.05, respectively. A separate 2 × 2 repeated-measures ANOVA showed no main effect for either side or time following left cathodal stimulation (*p* > 0.05) (Fig. 2b, c).

To conclude, handedness-related vestibular dominance does concern both lower [11, 13, 14] and higher order vestibular function [7–10]. However, this does not detract from the hypothesis presented by Dieterich and Brandt, which focuses on the independent development in the child of hand dexterity and vestibular function [12]. Experimental and developmental studies in the future will have to examine whether data supports such a hypothesis. The task will be exciting but challenging given the widespread property of the human brain to lateralise large functional modules such as language, hand dexterity and spatial attentional/navigational functions that we now know also include vestibular cortical processing. Incidentally, a typographical error in the Brandt and Dieterich letter incorrectly states that language is lateralised to the right hemisphere!
